# Relationship between anal functional lumen imaging probe (EndoFLIP®) results and the clinical presentation of faecal incontinence

**DOI:** 10.1111/codi.16225

**Published:** 2022-07-19

**Authors:** Charlotte Desprez, Guillaume Gourcerol, Céline Savoye‐Collet, Valérie Bridoux, Thomas Duflot, Anne‐Marie Leroi

**Affiliations:** ^1^ Department of Digestive Physiology Rouen University Hospital Rouen France; ^2^ Department of Digestive Physiology, CIC‐CRB 1404, Inserm U1073, CHU Rouen 'Normandie Université, UNIROUEN Rouen France; ^3^ Department of Radiology 'Normandie Université, UNIROUEN, CHU Rouen Rouen France; ^4^ Department of Digestive Surgery, Inserm U1073 'Normandie Université, UNIROUEN, CHU Rouen Rouen France; ^5^ Department of Pharmacology 'Normandie Université, UNIROUEN, Inserm U1096, CHU Rouen Rouen France

## Abstract

**Aim:**

Faecal incontinence (FI) subtypes (urge, passive, mixed) are linked to the physiopathological mechanism of FI. Previous studies have failed to demonstrate a consistent relationship between FI subtype and anal sphincter dysfunction. Our aim was to evaluate the relationship between anal sphincter function, assessed using the new EndoFLIP® technology, and FI subtype.

**Method:**

Patients referred for FI were prospectively enrolled between October 2015 and May 2021 in a registry, and data were retrospectively examined. Each patient underwent a clinical assessment as well as three‐dimensional high‐resolution or water‐perfused anorectal manometry, anal EndoFLIP®, and anorectal electrophysiological and endoanal ultrasound tests. The results of the investigations were compared across FI subtypes.

**Results:**

The cohort included 133 patients, 54 (41%) of whom met the criteria for urge FI, 40 (30%) for passive FI and 39 (29%) for mixed FI. The resting anal distensibility index (DI) at 50 ml of distension was significantly lower in patients with urge FI than in patients with passive FI (*p* = 0.04). At rest, a DI at 50 ml of distension ≥7.3 mm^2^ mmHg^−1^ and a DI at 40 ml of distension <1.3 mm^2^ mmHg^−1^ were associated with the passive and urge FI subtypes, respectively, with poor discriminatory power (an accuracy of 0.49 compared with 0.33 for random assignment). There were no differences in anorectal manometry, endoanal ultrasound or electrophysiological test results among the urge, passive and mixed FI subgroups (all *p* > 0.05).

**Conclusion:**

The anal sphincter DI using the EndoFLIP® system displayed poor predictive performance in distinguishing among FI subtypes.

## What does this paper add to the literature?

The relationship between faecal incontinence (FI) subtype and anal sphincter dysfunction has not been clearly elucidated using standard tests. The new EndoFLIP® system for measuring anal distensibility can distinguish between FI subtypes in some extreme cases but is not powerful enough to be used alone to diagnose a given FI subtype.

## INTRODUCTION

Faecal incontinence (FI) is defined by the Rome IV criteria as the involuntary loss of faeces at least twice a month over a 6 month period [1]. This condition is classified by the International Continence Society (ICS) into two subtypes: urge incontinence, defined as the unwanted loss of stool despite active attempts to inhibit defaecation, and passive incontinence, defined as unwanted loss of stool without patient awareness [2]. However, a third subtype, mixed FI, is also described in many studies and is defined as a combination of urge and passive FI [3]. This subtype may be related to the underlying pathophysiological mechanisms responsible for FI and may serve as a guide for first‐line therapeutics [4]. In clinical practice, dysfunction of the external anal sphincter leading to decreased squeeze pressures is associated with urge FI [5, 6, 7] while impairment of the internal anal sphincter has been linked to passive incontinence [5, 8]. Previous studies have not demonstrated a clear association between FI subtype and anal sphincter dysfunction [3]. This could be due to a lack of sensitivity of current anorectal investigations (i.e. manometry) for properly assessing anal sphincter function [9].

The EndoFLIP® system was developed to study a new parameter of anal sphincter competence, i.e. sphincter resistance to transphincteric flow or the distensibility index (DI) [10]. The DI is abnormally high in patients with FI [11, 12, 13, 14] and better discriminates healthy subjects from patients with FI than manometric anal pressures [13]. The anal DI could thus be a prime determinant of sphincteric strength [15].

The aim of the present study was to evaluate the relationship between FI subtype and the anal DI evaluated using EndoFLIP® technology.

## METHOD

### Patients

Patients with FI who were referred to the Physiology Unit of Rouen University Hospital (France) between October 2015 and May 2021 for physiological investigations of anorectal function were prospectively enrolled in a registry, and their data were retrospectively examined. Age (below 18 years) and isolated gas incontinence were the only exclusion criteria. As our objective was to assess the relationship between FI subtypes and their mechanisms using EndoFLIP® technology, we wanted to explore a large population of FI patients with no criteria of selection for age or sex to avoid selection bias with respect to the aetiology of the sphincter deficiency (e.g. obstetric trauma could be more frequently observed in younger women) and to evaluate EndoFLIP® in the routine practice of a tertiary care centre.

Some of the patients had been included in a previous study [14]. Ethical approval for data collection from patients with FI was obtained in accordance with data protection and civil liberties legislation (CNIL, E2022‐07).

### Clinical assessment of FI patients

During the screening visit, the clinical characteristics of the patients were recorded. Additional information, including duration of FI, details of stool consistency using the Bristol stool form scale [16] and the presence of transit disorders [17] (Rome IV criteria) was collected. FI severity was assessed using the Cleveland Clinic Incontinence Score (CCIS) [18] and the Vaizey score [19]. The Vaizey score considers the same items as the CCIS in addition to items concerning the use of constipating medication and the lack of ability to defer defaecation for 15 min [19]. Quality of life (QOL) was assessed using the French version of the American Society of Colon and Rectal Surgeons QOL auto‐questionnaire for FI (FIQL) [20]. One of the items of the embarrassment domain of the FIQL questionnaire is related to passive FI (‘I leak stool without even knowing it.’) [20].

To classify patients according to FI subtype, definitions validated by the ICS were used in the present study [2]. Urge incontinence was defined as the unwanted loss of stool despite active attempts to inhibit defaecation. Passive incontinence was defined as the unwanted loss of stool without the person being aware. This information was collected during structured interviews that were not standardized and/or validated conducted in our routine practice by three trained physicians from our department (AML, VB, GG). Patients who said they were almost exclusively incontinent because they had ‘great urgency and could not reach the toilet on time’ were considered to have urge incontinence whereas patients who said they were almost exclusively ‘unaware when the leakage was actually happening’ were considered to have passive incontinence. Lastly, patients who reported both FI subtypes were considered to have mixed incontinence. Three subgroups of patients were created based on FI subtype (urge, passive, mixed).

### Anorectal functional tests

Each FI patient underwent a clinical examination and a battery of pelvic floor examinations, including three‐dimensional high‐resolution anorectal manometry (3D‐HRAM) or water‐perfused anorectal manometry (depending on the time period), as well as electrophysiological and endoanal ultrasound tests. An anoscopy–rectoscopy was performed to assess anorectal intussusception and prolapse. Water‐perfused anorectal manometry and 3D‐HRAM, endoanal ultrasounds, electrophysiological tests and anoscopy–rectoscopy were performed as previously described [14, 21, 22].

Regardless of the type of manometry used (3D‐HR or water‐perfused), manoeuvres were performed with a standard sequence that included a 30 s recovery period between each manoeuvre: rest—anorectal pressures were measured with the subject relaxed after a 15 min rest period; squeeze—the subject was instructed to squeeze the anal canal as strongly and for as long as possible (two attempts). The best performance of the two trials was selected and used for the analysis. The rectal sensory test involved inflating a rectal balloon with air in 10 ml increments using a hand‐held syringe and recording the threshold volumes and pressures for minimal sensation and maximum tolerated volume [14].

The likely underlying mechanisms of FI were determined based on clinical assessment, anorectal manometry, EndoFLIP® and anoscopy–rectoscopy results. Nine possible isolated or associated underlying mechanisms for FI were identified for each patient: anal hypocontractility (abnormal maximal voluntary squeeze pressure and/or duration), anal hypotonia (abnormal anal resting pressure), rectal hypersensitivity (reduced rectal capacity with or without low compliance), rectal hyposensitivity (elevated rectal capacity with or without high compliance), constipation, diarrhoea, procidencia/prolapse, low anterior resection syndrome (LARS) and idiopathic. As the subtype of FI is supposed to direct us toward an anorectal dysfunction without prejudging its aetiology, we only listed the different types of anorectal dysfunctions but not the potential causes of these dysfunctions (i.e. anal sphincter defect, pudendal neuropathy).

### EndoFLIP® assessments

The DI of the anal sphincter was measured using the EndoFLIP® system (Crospon Ltd) as previously described [13]. The device (catheter EF‐325 N) consists of a two‐lumen polyethylene tube with an outer diameter of 3 mm. Seventeen ring electrodes placed at 4 mm intervals measured electrical impedance to estimate the cross‐sectional area (CSA) at 16 points, 5 mm apart. The CSA measurements were performed over an 8 cm long zone. A 12 cm long bag was mounted on the probe and was filled with saline solution to a maximum diameter of 25 mm. The intra‐bag pressure was calculated using a solid‐state pressure transducer placed inside the bag at the distal end. During the procedure, the EndoFLIP® probe was inserted and two detection electrodes remained visible outside the anal verge. The probe was held in place manually. The bag was filled with 0–50 ml of conductive saline solution in 10 ml increments at a rate of 40 ml/min. The resting measurements were recorded after 30 s, following which the subjects were asked to squeeze for each inflation level.

### Statistical analyses

The concurrent validity, including sensitivity, specificity and positive and negative predictive values of the two items related to faecal urgency in the Vaizey score [19] and to passive FI in the FIQL questionnaire [20] were analysed and were compared with the medical interview as a reference standard.

The EndoFLIP® and 3D‐HRAM data were analysed as described elsewhere [13]. Sixteen CSAs and intra‐bag pressures were assessed with 40 ml and 50 ml of saline solution at rest and during voluntary contractions. The DI (mm^2^ mmHg^−1^) was defined as the CSA at the narrowest point divided by the corresponding intra‐bag pressure at rest and during peak voluntary contraction. As we have previously shown that the 40 ml and 50 ml DI at rest and during squeeze are the most relevant parameters for exploring patients with FI [13], we only used these indices in the present study. For anorectal manometry, the following parameters were analysed: length of the high‐pressure zone (for 3D‐HRAM), resting pressure (eSleeve resting pressure for 3D‐HRAM), incremental maximal and mean squeeze pressures (maximal/mean squeeze pressure minus resting pressure), duration of the sustained anal contraction, rectal sensation, volumes and compliance [14].

Given the different manometric systems (high‐resolution and water‐perfused) with specific normal values, the manometry and EndoFLIP® measurements were also expressed as normal or abnormal sphincter/rectal function. The cut‐off values for the DIs, pressures and volumes used to divide the patients into normal and abnormal groups were obtained from a study of a normal healthy population of women for the 3D‐HRM and EndoFLIP® [13] measurements (10th–90th percentiles) and from our unpublished data for water‐perfused anorectal manometry (Table 1).

**TABLE 1:** Cut‐off values for the pressures, volumes, compliance and DIs used to divide patients into two groups (normal and abnormal) obtained from a study on a normal healthy population of women for 3D‐HRAM and EndoFLIP® (Gourcerol, 2016; 10th–90th percentiles) and from our unpublished data for water‐perfused anorectal manometry

Parameters	Normal values
Water‐perfused anorectal manometry	
Anal resting tone (cmH_2_O)	≥60
Maximum squeeze pressure (cmH_2_O)	≥60
Threshold volume (ml)	≤20
Maximal tolerated volume (ml)	100–400
Rectal compliance (ml cmH_2_O^−1^)	2–10
3D‐HRAM	
Anal resting tone (mmHg)	>58
Maximum squeeze pressure (mmHg)	>54
Mean squeeze pressure (mmHg)	>6
Squeeze pressure duration (s)	>15
Threshold volume (ml)	>30
Maximal tolerated volume (ml)	150–340
Rectal compliance (ml mmHg^−1^)	5–12
Anal EndoFLIP®	
40 ml DI at rest (mm^2^ mmHg^−1^)	<1
40 ml DI during squeeze (mm^2^ mmHg^−1^)	<0.6
50 ml DI at rest (mm^2^ mmHg^−1^)	<2.9
50 ml DI during squeeze (mm^2^ mmHg^−1^)	<1.5

*Abbreviations:* 3D‐HRAM, three‐dimensional high‐resolution anal manometry; DI, distensibility index.

The FI subtypes were compared between patients with a majority of FI mechanisms known to provoke urge incontinence (anal hypocontractility, hypersensitive rectum, diarrhoea) [7, 23] and those with a majority of mechanisms known to provoke passive FI (anal hypotonia, hyposensitive rectum, procidentia, constipation) [24, 25, 26].

Continuous and count data were expressed as medians [interquartile range, IQR] and *n* (%), or *n*/*N* (%), respectively, where *N* is the total number of patients with available data. The *p*‐values were computed using the chi‐square test or Fisher's exact test for nominal data and analysis of variance (ANOVA) for continuous data. A post hoc pairwise comparison test was performed when the values of Fisher's exact test were significant, with corrections for multiple testing (false discovery rate, Benjamini–Hochberg). Tukey's honestly significant difference method was used for nominal and continuous data. The FI subtype classification tree was built based on the EndoFLIP® results using the recursive partitioning method. Hyperparameters were optimized by machine learning.

Data analyses, statistical analyses and graph plotting were performed using R v.4.1.0 [27], *rstatix* [28], *caret* [29], *ggplot2* [30], *ggsci* [31], *ggpubr* [32], rpart [33], *rpart.plo* [34], and *mlr* [35]. Raw data as well as the R code are available as Supplementary Material S1 and S2, respectively. Our study meets the criteria of the Strobe List (Appendix S1)

## RESULTS

### Clinical characteristics of patients

One hundred thirty‐three patients were consecutively included in the present study (median age 64 [55–73] years; M:F ratio 14:119). Patient characteristics are summarized in Table 2. After an interview, 54 (41%) patients were assigned to the urge FI subgroup, 40 (30%) to the passive FI subgroup and 39 (29%) to the mixed FI subgroup. The items of the scores concerning the presence of urge FI (faecal urgency with the Vaizey score) and passive FI (stool leakage without awareness with the FIQL score) showed fairly good sensitivity but poor specificity for discriminating the FI subtype compared with the medical interview, which was the reference standard (Table 3).

**TABLE 2:** Clinical characteristics of the 133 patients with FI according to subtype

Parameter	All (*N* = 133)	Urge FI (*N* = 54)	Passive FI (*N* = 40)	Mixed FI (*N* = 39)	*p*‐value
Age (years)	64 [55–73]	64.5 [55.5–71.5]	70 [59–76]*	63 [50–69]*	0.014
Sex (male/female)	14/119	5/49	6/34	3/36	0.593
BMI (kg m^−2^)	26.3 [23.1–29.3]	27.0 [22.7–29.7]	24.8 [22.1–27.4]	26.6 [24.1–29.6]	0.143
Parity	2.2 [1.8–3]	2.0 [2.0–3.0]	2 [2.0–3.0]	2 [1.0–2.5]	0.127
Medical comorbidities, *n* (%)					
Neurological disease	17 (12.8)	4 (7.4)	6 (15)	7 (17.9)	0.285
Psychiatric disease	18 (13.3)	5 (9.3)	6 (15.0)	7 (17.9)	0.457
Anorectal surgery	39 (29.3)	12 (22.2)**	8 (20.0)*	19 (48.7)**^,^*	0.006
Diabetes	12 (9.0)	8 (14.8)	3 (7.5)	1 (2.6)	0.115
Colorectal cancer	4 (3.0)	1 (1.9)	0 (0.0)	3 (7.7)	0.139
Irritable bowel syndrome	37 (28.7)	9 (17.3)	14 (35.9)	14 (36.8)	0.063
Menopause	89 (73.6)	36 (73.5)	30 (85.7)	23 (62.2)	0.077
Traumatic delivery	72 (59.5)	32 (65.3)	16 (45.7)	24 (64.9)	0.143
Associated urinary incontinence	40 (30.1)	14 (25.9)	15 (37.5)	11 (28.2)	0.665
Transit disorders, *n* (%)					
Diarrhoea	30 (22.5)	15 (27.8)	5 (12.5)	10 (25.6)	0.185
Constipation	21 (15.8)	2 (3.7)***	17 (42.5)*^,^***	2 (5.1)*	0.0001
Duration, *n* (%)	***N* = 130**	***N* = 52**	***N* = 39**	***N* = 39**	
<1 year	18 (13.8)	6 (11.5)	6 (15.4)	6 (15.4)	
1–5 years	57 (43.8)	21 (40.4)	21 (53.8)	15 (38.5)	
5–10 years	29 (22.3)	13 (25.0)	7 (17.9)	9 (23.1)	
>10 years	26 (22.0)	12 (23.1)	5 (12.8)	9 (23.1)	
CCIS	11 [8–14]	11 [8–14]	11 [8–14]	12 [10–15]	0.073
Vaizey score	14 [11–17]	14 [12–16]	12 [10–16]*	15 [13–18]*	0.009
FIQL					
Lifestyle	2.6 [1.8–3.1]	2.7 [1.7–3.1]	2.7 [2.3–3.3]*	2.3 [1.5–3.0]*	0.048
Coping and behaviour	1.7 [1.3–2.3]	1.7 [1.2–2.1]***	1.9 [1.4–2.8]*^,^***	1.4 [1.2–2.2]*	0.007
Depression and self‐perception	2.6 [1.9–3.3]	2.7 [2.0–3.2]	2.7 [2.1–3.4]	2.1 [1.8–3.3]	0.348
Embarrassment	2.0 [1.3–2.7]	2.0 [1.7–2.7]**	2.0 [1.3–2.7]	1.7 [1.2–2.0]**	0.020
Bristol stool form scale^a^	*N* = 129	*N* = 52	*N* = 39	*N* = 38	‐
Type I–II	32 (24.8%)	14 (26.9%)	12 (30.8%)	6 (15.8%)	0.283
Type III–IV–V	65 (50.4%)	21 (40.4%)	20 (51.3%)	24 (63.2%)	0.102
Type VI–VII	50 (38.8%)	24 (46.2%)	13 (33.3%)	13 (34.2%)	0.366
Wearing a protective pad	*N* = 130	*N* = 52	*N* = 39	*N* = 39	
Never	28 (21.5%)	15 (28.8%)	8 (20.5%)	5 (12.8%)	0.299
<1 per week	14 (10.8%)	7 (13.5%)	2 (5.1%)	5 (12.8%)	
>1 per week	15 (11.5%)	4 (7.7%)	7 (17.9%)	4 (10.3%)	
Every day	73 (56.2%)	26 (50.0%)	22 (56.4%)	25 (64.1%)	
Endoanal ultrasonography	*N* = 114	*N* = 47	*N* = 32	*N* = 35	
External anal sphincter defect	31 (27.2%)	15 (31.9%)	5 (15.6%)	11 (31.4%)	0.198
Internal anal sphincter defect	33 (28.9%)	14 (29.8%)	6 (18.8%)	13 (37.1%)	0.240
Anorectal electrophysiological tests	*N* = 99	*N* = 45	*N* = 25	*N* = 29	
Abnormal tests	59 (59.6%)	25 (55.6%)	18 (72%)	16 (55.2%)	0.331

*Note:* Data are expressed as medians [IQR], *n* (%), or *n*/*N* (%), where *N* is the total number of patients with available data. *p*‐values were computed using the chi‐square test or Fisher's exact test for nominal data and analysis of variance for continuous data. Post hoc pairwise comparison tests were performed when the Fisher's exact test values were significant. Tukey's honestly significant difference method was used for nominal and continuous values.

*Abbreviations:* BMI, body mass index; CCIS, Cleveland Clinic Incontinence Score; FI, faecal incontinence; FIQL, Faecal Incontinence Quality of Life.

*p < 0.05 passive FI vs. mixed FI.

**p < 0.05 urge FI vs. mixed FI.

***p < 0.05 urge FI vs. passive FI.

^a^ Some patients may belong to several groups.

**TABLE 3:** Sensitivity, specificity, positive and negative predictive values and Youden's index of each item related to urge FI of the Vaizey score and passive FI of the FIQL score compared to the medical interview, which was the reference standard, to classify patients into the urge or passive FI subgroup

	Sensitivity (%)	Specificity (%)	Youden's index (%)	PPV (%)	NPV (%)
Item urgency (Vaizey score)	92	23	15	61.5	69
Item passive FI (FIQL score)	87	23	9	46	70.6

*Abbreviations:* FI, faecal incontinence; FIQL, Faecal Incontinence Quality of Life; NPV, negative predictive value; PPV, positive predictive value;.

A comparison of patient characteristics in the different subgroups is shown in Table 2. Patients with passive FI were older than patients with mixed FI (*p* = 0.01; Table 2). More patients with mixed FI underwent anorectal surgery (rectopexy, proctectomy, haemorrhoid surgery, fissurectomy, treatment of anal fistula) than patients with urge or passive FI (*p* < 0.001; Table 2). Constipation occurred more frequently in patients with passive FI than in the two other subgroups (*p* < 0.001; Table 2).

The FIQL lifestyle and coping scores were lower in patients with mixed FI than in patients with passive FI (*p* = 0.048 and *p* = 0.007, respectively; Table 2). The FIQL coping score was lower in patients with urge FI than in patients with passive FI (*p* = 0.007: Table 2). The FIQL embarrassment score was lower in patients with mixed FI than in those with urge FI (*p* = 0.020; Table 2). The Vaizey score was higher in patients with mixed FI than in patients with passive FI (*p* = 0.009; Table 2).

### Anorectal functional tests

#### Anorectal manometry

There was no difference between FI subtypes with respect to the parameters measured with either HRAM or water‐perfused anorectal manometry, even after pooling the patients based on normal/abnormal results (Table 4).

**TABLE 4:** Results of perfused anorectal manometry and high‐resolution anorectal manometry and the proportion of abnormal findings using both methods according to FI subtype

Parameters	Urge FI	Passive FI	Mixed FI	*p*‐value
Water‐perfused anorectal manometry	*N* = 22	*N* = 15	*N* = 17	
Maximal resting anal pressure (cmH_2_O)	56.0 [34.8–70.8]	35.0 [23.5–77.5]	45.0 [37.0–60.0]	0.776
Maximal VC amplitude (cmH_2_O)	29.0 [19.3–46.0]	46.0 [10.0–97.5]	23.0 [15.0–80.0]	0.708
Mean VC amplitude (cmH_2_O)	17.0 [11.0–24.0]	10.0 [0.0–26]	15.0 [10.0–27.0]	0.998
Duration of VC (s)	18.5 [5.3–40.8]	10.0 [1.5–42.5]	15.0 [6.0–47.0]	0.829
First sensation volume (ml)	10 [10–10]	10 [10–10]	10.0 [10.0–10.0]	0.893
Constant sensation volume (ml)	100 [81–119]	88 [50–138]	75 [44–125]	0.452
Maximum tolerated volume (ml)	150 [125–175]	125 [100–188]	150 [75–200]	0.813
Compliance (ml cmH_2_O^−1^)	3.4 [2.4–4.4]	3.2 [1.7–4.8]	3.1 [2.1–4.0]	0.284
High‐resolution anorectal manometry	*N* = 32	*N* = 25	*N* = 22	
Maximal resting anal pressure (cmH_2_O)	52.6 [34.3–64.8]	38.0 [29.0–49.0]	38.0 [28.0–53.8]	0.117
Maximal VC amplitude (cmH_2_O)	49.5 [26.8–68.3]	62.0 [40.0–110.0]	42.5 [26.3–75.5]	0.479
Mean VC amplitude (cmH_2_O)	10.0 [5.0–18.0]	13.0 [9.5–19.0]	12.0 [5.0–26.0]	0.992
Duration of VC (s)	19.0 [5.0–44.3]	15.0 [6.0–44.0]	40.0 [12.0–44.0]	0.543
First sensation volume (ml)	15 [10–30]	20 [10–30]	10 [10–20]	0.181
Constant sensation volume (ml)	100 [75–125]	100 [75–138]	125 [75–150]	0.353
Maximum tolerated volume (ml)	150 [125–200]	150 [125–225]	175 [150–206]	0.383
Compliance (ml cmH_2_O^−1^)	4.7 [3.8–6.1]	6.5 [5.0–8.4]	6.8 [4.9–9.6]	0.463
Overall	*N* = 54	*N* = 40	*N* = 39	
Abnormal resting anal pressure	32 (59.2%)	31 (77.5%)	30 (76.9%)	0.087
Abnormal maximal VC amplitude	37 (68.5%)	18 (45.0%)	25 (64.1%)	0.059
Abnormal VC duration	25 (46.3%)	23 (57.5%)	15 (38.5%)	0.560
	*N* = 47	*N* = 32	*N* = 36	
Rectal hypersensitivity	18 (33.3%)	15 (46.9%)	12 (33.3%)	0.512
Rectal hyposensitivity	2 (4.2%)	2 (6.2%)	2 (5.5%)	0.921

*Note:* Data are expressed as median [IQR], *n* (%) or *n*/*N* (%), where *N* is the total number of patients with available data.

*p*‐values were computed using the chi‐square test or Fisher's exact test for nominal data and analysis of variance for continuous data. Post hoc pairwise comparison tests were performed when the Fisher's exact test values were significant. Tukey's honestly significant difference method was used for nominal and continuous values.

Abbreviations: FI, faecal incontinence; VC, voluntary contraction.

#### EndoFLIP®

There was no difference between FI subtypes for the DI at 40 ml of distension at rest and during voluntary contraction (Table 5). The DI at rest at 50 ml of distension was lower in patients with urge FI than in patients with passive FI (*p* = 0.046; Table 5), but not during voluntary contraction (Table 5).

**TABLE 5:** Results of EndoFLIP® measurements according to FI subtype in a cohort of 133 patients

Parameters	Urge FI (*N* = 54)	Passive FI (*N* = 40)	Mixed FI (*N* = 39)	*p*‐value
DI at rest, 40 ml of distension (mm^2^ mmHg^−1^)	1.92 [0.70–2.53]	1.85 [0.68–3.09]	1.50 [1.15–2.55]	0.121
Abnormal, *n* (%)	35 (64.8)	27 (67.5)	31 (79.5)	0.274
DI during VC, 40 ml of distension (mm^2^ mmHg^−1^)	0.95 [0.50–1.48]	0.75 [0.40–1.75]	1.10 [0.40–1.30]	0.937
Abnormal, *n* (%)	38 (70.4)	25 (62.5)	28 (71.8)	0.626
DI at rest, 50 ml of distension (mm^2^ mmHg^−1^)	2.25 [1.40–4.83]*	3.45 [1.83–6.78]*	3.20 [1.95–4.80]	0.046
Abnormal, *n* (%)	19 (35.2)	22 (55)	22 (56.4)	0.064
DI during VC, 50 ml of distension (mm^2^ mmHg^−1^)	1.70 [1.10–2.83]	2.19 [1.00–2.60]	2.08 [0.95–2.80]	0.924
Abnormal, *n* (%)	28 (51.9)	23 (57.5)	24 (61.5)	0.844

*Note:* Data are expressed as medians [IQR], *n* (%) or *n*/*N* (%), where *N* is the total number of patients with available data.

*p*‐values were computed using the chi‐square test or Fisher's exact test for nominal data and analysis of variance for continuous data. Post hoc pairwise comparison tests were performed when the Fisher's exact test values were significant. Tukey's honestly significant difference method was used for nominal and continuous values.

Abbreviations: DI, Distensibility Index; FI, faecal incontinence; VC, voluntary contraction.

*p < 0.05 urge FI vs. passive FI.

**FIGURE 1:**
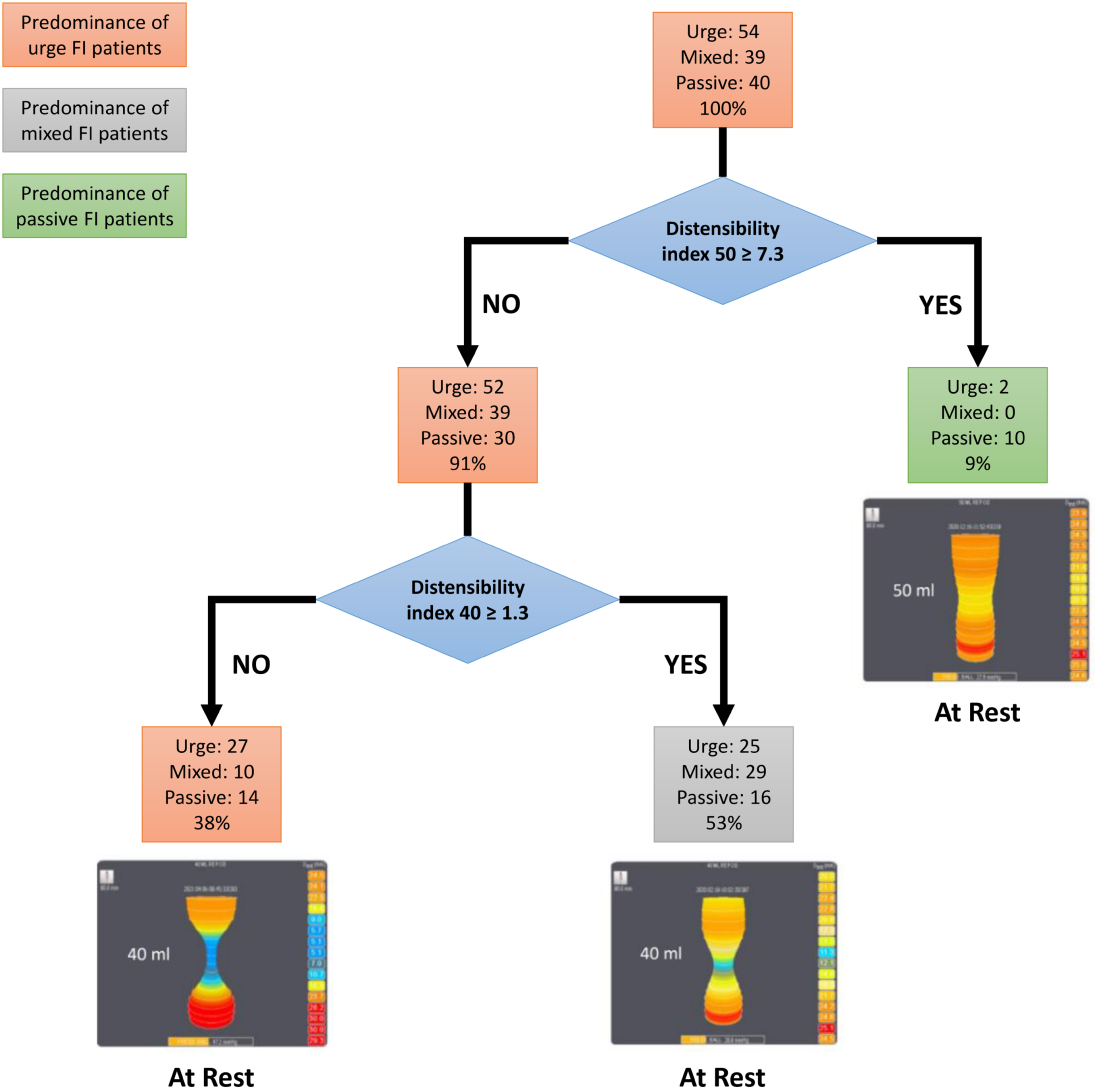
Classification tree of faecal incontinence (FI) subtypes based on EndoFLIP® measurements at rest. Values inside nodes correspond to the number of patients with urge, mixed or passive FI, and the corresponding percentage of total patients (lower value). The predominant type of FI is given for each box using colours (orange for urge FI, grey for mixed FI and green for passive FI). For each distensibility index, an example of an EndoFLIP® measurement is given. FI, faecal incontinence

A classification tree was built to determine whether DI thresholds at 40 or 50 ml of distension could predict FI subtype (Figure 1). At rest, a DI at 50 ml of distension ≥7.3 mm^2^ mmHg^−1^ identified patients with passive FI and a DI at 40 ml of distension <1.3 mm^2^ mmHg^−1^ distinguished urge FI from mixed FI. The accuracy of the classification tree was 0.49 (95% CI 0.41–0.58). The sensitivity, specificity and positive and negative predictive values of the classification tree to detect FI subtypes are given in Table 6.

**TABLE 6:** Performance parameters of the classification tree given in Figure 1 for detecting FI subtypes using distensibility index (DI) thresholds at 40 and 50 ml of distension

	Urge FI	Mixed FI	Passive FI
Sensitivity	0.53	0.41	0.83
Specificity	0.67	0.84	0.75
Positive predictive value	0.50	0.74	0.25
Negative predictive value	0.70	0.56	0.98

*Note:* At rest, a DI at 50 ml of distension ≥7.3 mm^2^ mmHg^−1^ identified patients with passive FI and a DI at 40 ml of distension <1.3 mm^2^ mmHg^−1^ distinguished urge FI from mixed FI.

Abbreviation: FI, faecal incontinence.

#### Other tests

**FIGURE 2:**
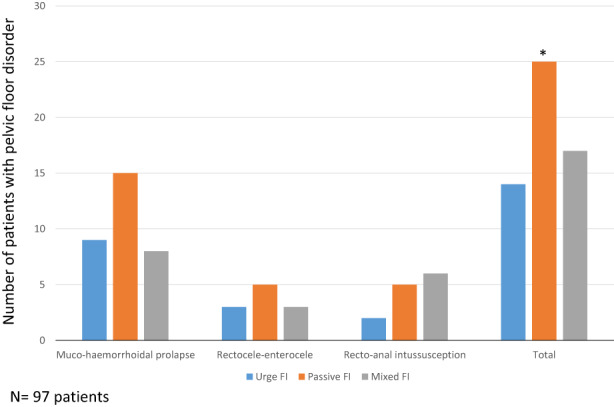
Pelvic floor disorders (recto‐anal intussusception, muco‐haemorrhoidal prolapse, rectocele, enterocele) found on anoscopy–rectoscopy in 97 patients based on faecal incontinence (FI) subtype. Some patients could have more than one disorder. *p < 0.05

One hundred fourteen patients underwent endoanal ultrasonography (86%). There was no difference between the different FI subtypes with respect to the presence of anal sphincter lesions for both internal and external anal sphincters (Table 2). Anorectal electrophysiological tests were performed in 99 patients (74%). There was no association between the anorectal electrophysiological test results (normal/abnormal) and the FI subtype (Table 2). Pelvic floor disorders on anoscopy–rectoscopy were found more frequently in patients with passive or mixed FI than in patients with urge FI (*p* = 0.005; Figure 2).

### Mechanisms of FI

**FIGURE 3:**
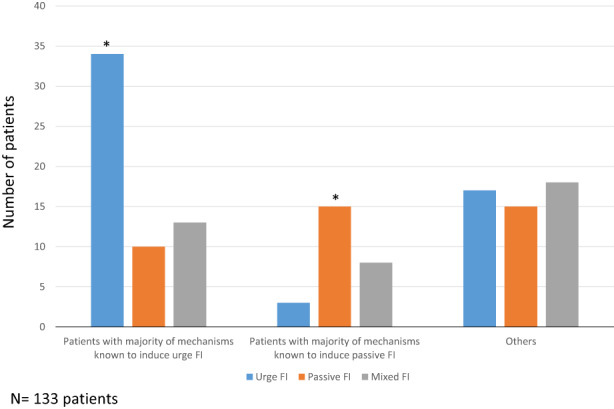
Relationship between the identified mechanisms of faecal incontinence (FI) in a cohort of 133 patients and FI subtype. *p < 0.05

One hundred and seven patients (80%) had several associated identified mechanisms that could explain their incontinence (Table 7). Patients with a predominance of identified mechanisms known to induce urge incontinence were significantly more represented in the urge FI subgroup (*p* = 0.0004; Figure 3). Similarly, patients with a predominance of identified mechanisms known to induce passive incontinence were significantly more represented in the passive FI subgroup (*p* = 0.0004; Figure 3).

**TABLE 7:** Underlying mechanisms of FI in a cohort of 133 patients complaining of FI

Identified FI mechanisms	No. of patients (*N* = 133)
Isolated mechanisms	
Anal hypocontractility	10
Anal hypotonia	9
Rectal hyposensitivity	1
Constipation	3
Idiopathic	3
Associated mechanisms	
Anal hypocontractility + anal hypotonia	
Isolated	23
With transit disorders	14
With transit disorders and procidencia	1
With procidencia	1
Anal hypocontractility + anal hypotonia + rectal hypersensitivity	
Isolated	15
With transit disorders	10
With procidencia	1
Anal hypocontractility + anal hypotonia + rectal hyposensitivity	
Isolated	1
With transit disorders	2
Anal hypocontractility + rectal hypersensitivity	
Isolated	5
With transit disorders	1
With procidencia	1
With transit disorders and procidencia	1
Anal hypocontractility + rectal hyposensitivity	
Isolated	2
With transit disorders	1
Anal hypocontractility + transit disorders	7
Anal hypotonia + rectal hypersensitivity:	
Isolated	4
With transit disorders	3
Anal hypotonia + rectal hyposensitivity	1
Anal hypotonia + transit disorders	5
Anal hypotonia + procidencia	1
Rectal hypersensitivity + transit disorders	1
Rectal hyposensitivity + transit disorders	1
LARS	5

Abbreviations: FI, faecal incontinence; LARS, low anterior resection syndrome.

## DISCUSSION

We studied the relationship between anal DI and FI subtype using the EndoFLIP® system. A weak relationship was detected between the anal DI at rest at 50 ml of distension and passive FI. A dysfunction of the internal anal sphincter may lead to poor sealing of the anal canal under rest, which may cause passive FI. A dysfunction of the external anal sphincter may lead to insufficient closure of the anal canal when stool arrives in the rectum, which may cause urge incontinence [1]. Given these two mechanisms, we expected to correlate urge FI with external anal sphincter dysfunction and passive FI with internal anal sphincter dysfunction. However, as previously reported, we did not find any significant difference in the distribution of anal hypocontractility and anal hypotonia recorded by anal manometry between the three FI subtypes [6, 36, 37, 38]. The frequency of anatomical and/or neuropathic sphincter lesions was not related to a particular FI subtype, as expected, as this represented more the potential cause of the dysfunction than the dysfunction itself. Given the superiority of the EndoFLIP® system in quantifying resistance to anal distension, an important variable for anal continence [10, 12, 39], it should be more accurate in detecting sphincter dysfunctions. In the present study, the resting anal DI at 50 ml of distension differed significantly, with the passive subtype having the highest values and the urge subtype the lowest. However, anal DI during voluntary contraction was not significantly different between the FI subtypes. The DI at squeeze may more reflect the isolated action of the external anal sphincter while the DI at rest may more reflect the isolated action of the internal anal sphincter [13]. We identified the values of the DIs that allowed us to discriminate patients according to their FI subtype in a descriptive manner. We used a classification tree to show that patients with a resting DI at 50 ml of distension ≥7.3 mm^2^ mmHg^−1^ and that resting DIs at 40 ml of distension <1.3 mm^2^ mmHg^−1^ were related to the passive and urge FI subtypes, respectively. However, the discriminatory power of the classification tree was low (an accuracy of 0.49 compared with 0.33 for random assignment). Although EndoFLIP® can distinguish between different FI subtypes, this is true only for extreme DI values.

The main premise for explaining discrepancies between anal sphincter function and FI subtype could be the lack of consensus in categorizing patients into the different subgroups. Other studies have used medical interviews, validated FI scores or rating scales [3]. The present study showed that these choices are very important given the poor agreement between the FI score results and the interviews conducted by a trained physician to classify patients according to FI subtype. In addition, we categorized patients based on the exclusivity of one type of FI compared with others. This made it possible to have more homogeneous groups of patients but had the disadvantage of creating smaller groups.

Lastly, our findings suggested that the most likely premise for explaining why FI subtype is moderately helpful in identifying the mechanism of incontinence is the multiple pathophysiological abnormalities identified in 80% of our patients. Sphincter problems are not the only reason for FI. Transit, pelvic floor disorders and rectal dysfunction may also be involved [7, 23, 24, 25, 26]. Although our results confirmed the association between the presence of passive FI, constipation and/or pelvic floor disorders, there were no statistically significant differences in the frequency of diarrhoea, abnormal rectal sensory volumes or compliance between the three subgroups of incontinent patients. The differences observed from other studies could be related to a declarative definition of diarrhoea or the non‐use of a barostat, a reference technique for assessing rectal capacity, sensitivity and compliance. However, when we identified the different FI mechanisms in each patient and distinguished patients in whom the mechanisms known to cause urge or passive FI were predominant, a significant relationship between FI subtype and the mechanisms was observed.

Although FI subtype appears insufficient to identify the exact mechanisms of FI, it remains useful for the evaluation and therapeutic management of incontinent patients. Like others [37], we observed that patients with urge or mixed FI have worse QOL scores than patients with passive FI. Patients with urge or mixed FI may thus benefit from rapid additional counselling on coping mechanisms for dealing with their symptoms.

The present study had some limitations. Although the data were prospectively collected, the analysis was retrospective. As explorations have changed with the arrival of HRAM, two types of anorectal manometries (water‐perfused and high‐resolution) were used, resulting in a loss of statistical power. Even dichotomizing patients on the basis of normal/anormal features did not allow us to precisely compare the performances of the manometry and EndoFLIP® techniques to predict FI subtype. Lastly, our definition of normal values for the DI, pressures and volumes was based on a preliminary study conducted only with women [13], unlike the present study that included a minority of men. However, the population of women studied was comparable in terms of parity with that of healthy subjects [13].

## CONCLUSION

In conclusion, the present study showed that the anal sphincter DI at rest can distinguish between FI subtypes, but with poor performance. The determination of the FI subtype appears inadequate for identifying the pathophysiological mechanisms of FI because of its frequent multiplicity. Although the determination of the FI subtype is interesting for evaluating patients, explorations that help identify the physiopathological mechanisms responsible for the FI and orientate the treatment are still required.

## AUTHOR CONTRIBUTIONS

All authors made substantial contributions to the conception and design of the study. CD and AML designed the research study and wrote the paper. CD, AML, GG and VB contributed to the inclusion of patients, CD and TD analysed the data, and CD, AML, TD, GG and VB contributed to the editing of the manuscript. All authors participated in drafting the manuscript or revising it critically for important intellectual content. They all gave final approval of the version to be submitted and of any revised versions.

## CONFLICT OF INTEREST

CD, VB and TD declare no conflicts of interest. AML provides expertise for Medtronic and gives oral presentations for Coloplast. GG gives oral presentations for Coloplast.

## ETHICAL STATEMENT

Ethical approval for data collection from patients was obtained in accordance with data protection and civil liberties legislation (CNIL, E2022‐07).

## Supporting information


Appendix S1

Material S1

Material S2

## Data Availability

Research data are not shared.
